# Asymptomatic and sub-microscopic *Plasmodium falciparum* infection in children in the Mount Cameroon area: a cross-sectional study on altitudinal influence, haematological parameters and risk factors

**DOI:** 10.1186/s12936-021-03916-7

**Published:** 2021-09-26

**Authors:** Irene Ule Ngole Sumbele, Rene Ning Teh, Gillian Asoba Nkeudem, Sorelle Mekachie Sandie, Marcel Nyuylam Moyeh, Robert Adamu Shey, Cabirou Mounchili Shintouo, Stephen Mbigha Ghogomu, Gaber El-Saber Batiha, Luay Alkazmi, Helen Kuokuo Kimbi

**Affiliations:** 1 Department of Zoology and Animal Physiology, University of Buea, Buea, Cameroon; 2 Department of Microbiology and Immunology, Cornell College of Veterinary Medicine, Ithaca, NY USA; 3 Department of Social Economy and Family Management, Higher Technical Teachers’ Training College, University of Buea, Kumba, Cameroon; 4 Department of Biochemistry and Molecular Biology, University of Buea, Buea, Cameroon; 5 Department of Pharmacology and Therapeutics, Damanhour University, Albeheira, Egypt; 6 Department of Biology, Faculty of Applied Science, Umm Al-Qura University, Mecca, Saudi Arabia; 7 Department of Medical Laboratory Science, The University of Bamenda, Bambili, Cameroon

**Keywords:** Anaemia, Asymptomatic malaria, Children, Haematological parameters, Sub-microscopic infection, *Plasmodium falciparum*, Risk factors, Cameroon

## Abstract

**Background:**

The Mount Cameroon area has experienced a 57.2% decline in confirmed malaria cases between 2006 and 2013 with the implementation of different control measures but, the disease is still of public health concern. The objective of the study was to assess the burden of asymptomatic and sub-microscopic *Plasmodium* infection, altitudinal influence on it, their effect on haematological parameters as well as identify the risk factors of infection.

**Methodology:**

A cross-sectional community-based survey involving 1319 children of both sexes aged 6 months to 14 years was conducted between July 2017 and May 2018. Malaria parasitaemia was confirmed by Giemsa-stained microscopy, sub-microscopic *Plasmodium* infection by 18S mRNA using nested PCR and full blood count analysis was done using an auto haematology analyser.

**Results:**

Malaria parasite, asymptomatic malaria parasitaemia and sub-microscopic *Plasmodium* infection and anaemia were prevalent in 36.4%, 34.0%, 43.8% and 62.3% of the children, respectively. The risk of having sub-microscopic *Plasmodium* infection was highest in children 5‒9 (OR = 3.13, P < 0.001) and 10‒14 years of age (OR = 8.18, P < 0.001), non-insecticide treated net users (OR = 1.69, P < 0.04) and those anaemic (OR = 9.01, P < 0.001). Children with sub-microscopic infection had a significantly lower mean haemoglobin (9.86 ± 1.7 g/dL, P < 0.001), red blood cell counts (4.48 ± 1.1 × 10^12^/L, P < 0.001), haematocrit (31.92%, P < 0.001), mean corpuscular haemoglobin concentration (313.25 ± 47.36, P = 0.035) and platelet counts (280.83 ± 112.62, P < 0.001) than their negative counterparts. Children < 5 years old (73.8%), having asymptomatic (69.8%) and sub-microscopic *Plasmodium* infection (78.3%) as well as resident in the middle belt (72.7%) had a higher prevalence of anaemia than their peers.

**Conclusion:**

The meaningful individual-level heterogeneity in the burden of asymptomatic and sub-microscopic *Plasmodium* infection in addition to its corollary on haematological variables among children in the different attitudinal sites of the Mount Cameroon Region accentuate the need for strategic context specific planning of malaria control and preventative measures.

**Supplementary Information:**

The online version contains supplementary material available at 10.1186/s12936-021-03916-7.

## Background

Globally, malaria is still a public health concern, although the death cases have steadily reduced from 736,000 in 2000 to 409,000 deaths in 2019. Cameroon accounts for 3% of this number [1], even with the recommended World Health Organization (WHO) control measures put in place [2]. In some malaria endemic areas, particularly Cameroon, where the disease burden is diverse, variations between altitudes and geographical areas [3, 4] may necessitate a strategic control measure. Falciparum malaria continues to negatively impact human life and in malaria-endemic countries, many *Plasmodium falciparum* infections manifest through various outcomes ranging from asymptomatic infection to the complicated disease, which may depend on the parasite density [5, 6] as well as the nutritional status of the host [3].

Asymptomatic malaria is defined by the WHO as the presence of asexual parasites in blood, without symptoms of illness [7]. Other studies defined asymptomatic malaria as the existence of malarial parasitaemia of any density in blood without any symptoms in individuals who have not received recent anti-malarial treatment in a given population [8]. This definition includes early detection of rising parasitaemia or any density of parasitized red blood cell (RBC) that is not enough to trigger a fever response [5].

Several studies in the central and East part of Africa have reported great numbers of asymptomatic malaria in endemic communities [9, 10]. In some of these communities, asymptomatic malaria parasite carriers represent a persistent pool for maintaining the life cycle and transmission of the *Plasmodium* species by the anopheline vector [11]. Also, Gouagna et al. [12] reported a higher tolerance of the malaria parasite among asymptomatic carriers when compared to symptomatic cases.

Sub-microscopic infections are present across different settings and populations [13, 14]. Okell et al. [15] reported that the prevalence of sub-microscopic infections is inversely correlated with slide prevalence and parasite density on the global level. Several reports in low transmission settings have also suggested higher proportions of sub-microscopic infections, when compared to microscopically detectable infections particularly in settings where recent malaria control efforts have been successful [5, 16]. This may not only be true in low transmission settings but may also be true in highly endemic communities, where a large proportion of asymptomatic infections may cause a partial non-sterilizing malaria immunity. Thus, it is necessary to uncover the extent of sub-microscopic infection in a high malaria transmission setting such as the Mount Cameroon area.

The existence of a significant sub-microscopic infection prevalence in an otherwise asymptomatic, microscopically negative population in Ethiopia [14] highlights that malaria infections can continue in a community even in the absence of illness. Nevertheless, although rarely causative agent of severe, acute symptoms, sub-microscopic malaria has been associated with several adverse outcomes during pregnancy [17], as well as mild anaemia [18] and various other symptoms (including coughing, vomiting, jaundice) in children under 10 years [19] in low endemic areas. However, little is known on its association with altitude and haematological indices especially in areas with moderate to high endemicities.

The Mount Cameroon Region represents a meso-hyperendemic setting and malaria epidemiology is highly heterogeneous as seen in many other parts of the country [4, 20]. This region has experienced a 57.2% malaria parasitaemia decline in confirmed malaria cases between 2006 and 2013 [21] mainly attributed to implementation of artemisinin-based combination therapy (ACT) and the wide scale distribution of long-lasting insecticidal nets (LLINs) [21]. In 2011, Cameroon distributed over eight million LLINs throughout the country [22]. The second nationwide distribution was carried out in 2016 and about 12 million LLINs were distributed. Yet, the disease prevalence is still a public health concern, and it remains unclear if this stability in endemicity over the years maybe associated with asymptomatic or sub-microscopic infections. However, there is no documented study relating the epidemiological characteristics of asymptomatic and sub-microscopic *Plasmodium* infection and their effects on haematological variables among children living in the different altitudinal sites along the Mount Cameroon area. Hence, determining the malaria parasite prevalence, its association with haematological variables in this endemic setting, which may contribute to clinical outcome and death will be fundamental for a strategic planning and control of morbidities associated with the infection.

## Methods

### Study area and participants

**Fig. 1:**
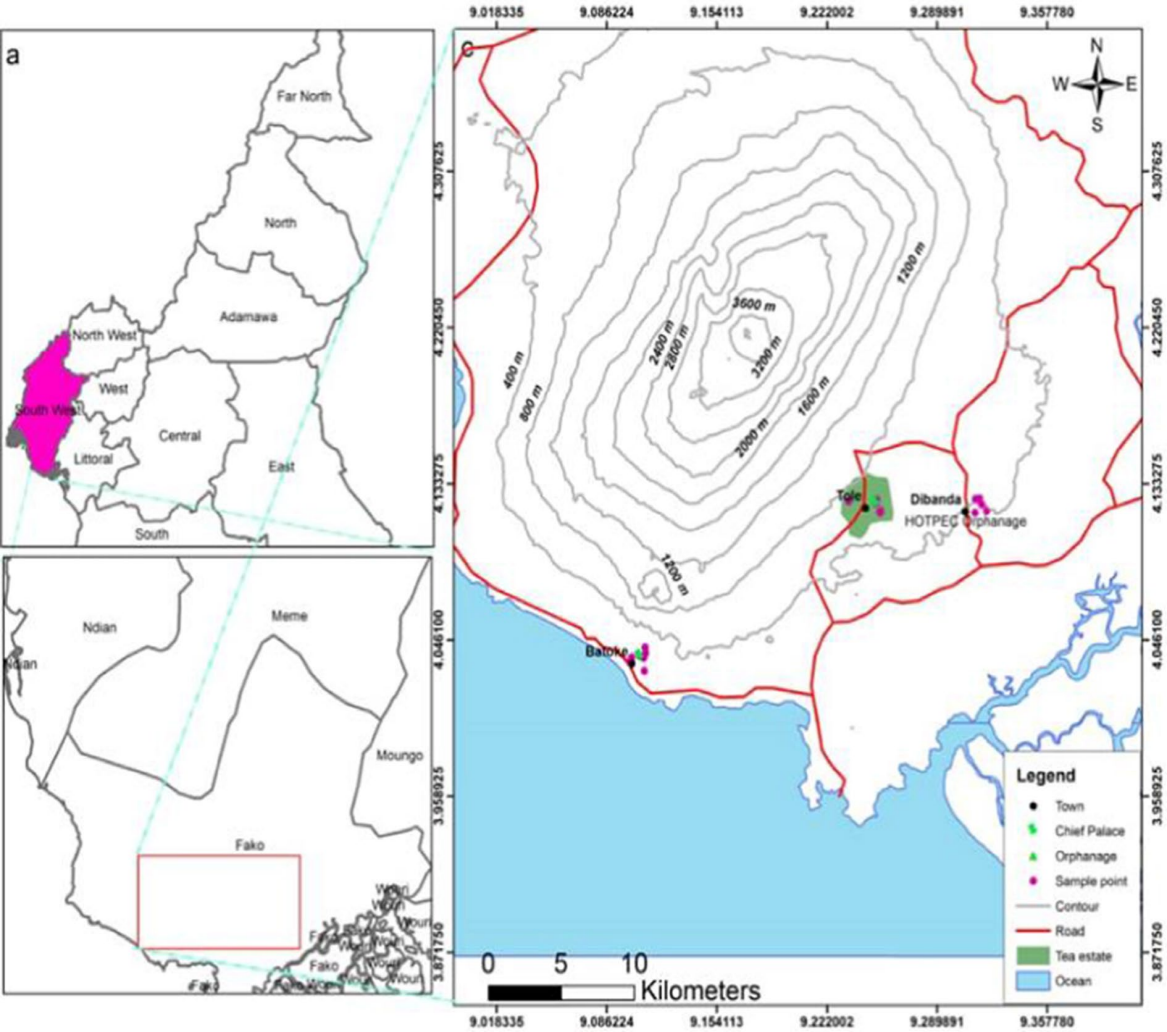
Location of the study sites in the Mount Cameroon area, Fako Division, South West Region, Cameroon

The study was carried out in Batoke (Limbe), Dibanda (Buea) and Tole (Buea), which are three semi-rural communities along different altitudinal ranges in the Mount Cameroon area, as shown in Fig. 1. The sites were classified as lowlands (< 200 m above sea level (asl)), middle belt (200–600 m asl) and highlands (> 600 m asl). The coordinates of Batoke range from altitude 8 m asl, latitude 04°01.364′ N, longitude 009°05.971′ E to 47 m, 04°02.039′ N and 009°05.808′ E; Dibanda from altitude 358 m asl, latitude 04°06.447′ N, longitude 009°18.725′ E to 400 m asl, 04°07.179′ N and 009°18.464′ E and Tole is located between 627 m asl, latitude 04°07.057′N, longitude 009°15.178′E and 630 m asl, latitude 04°6.906′N, longitude 09°14.434′E. These three study sites have been described in detail by Teh et al. [3, 23].

The work was conducted among pre- and school aged children of both sexes between 6 months and 14 years old, who granted assent and whose parents/caregivers consented to participate in the study. The inclusion and exclusion criteria of this study were revised according to Teh et al*.* [3].

### Study design

This cross-sectional community-based study was conducted in the rainy season between the months of July and October 2017 and between April and May 2018 in Tole, Batoke and Dibanda. Participants were invited for data collection in each community by their local chiefs and coordination was organised by the head/leader of a block within a neighbourhood (“quarter head”) of the various communities. Potential participants were reminded of the collection dates per block by the head/leader of the block. At the start of the study in each site, the parents, guardians and children were educated on the study protocol and the benefits of participation highlighted at their various neighbourhoods using an information sheet. Upon obtaining informed consent/assent from the parents/care givers, the study team proceeded with the collection of samples at specific identified collect sites.

### Sample size and sampling techniques

The sample size for each study altitude was calculated using the 66.2% prevalence of malaria in children in the study area [24]. Sample size was determined using the formula n = Z^2^pq/d^2^ [25] where n = the sample size required, z = 1.96: which is the standard normal deviate (for a 95% confidence interval, CI), p = 66.2%: proportion of malaria prevalence, q = 1—p: proportion of malaria negative children and d = acceptable error willing to be committed. The minimum sample size was estimated as n = 344 for each site. Considering a possible participation of more than one child per family, loss of samples due to blood clotting and incomplete data entry, the sample size was adjusted by 10% to a minimum of 379.

A multistage cluster sampling method was used to obtain the required sample. In the first stage, all the communities in the Mount Cameroon area were stratified into 3 zones namely lowland, Middlebelt and highland. One community was randomly selected from each zone namely, Tole (highland), Dibanda (middle belt) and Batoke (lowland). In the second stage, 21 clusters (quarters) were randomly selected from 31 clusters within the three communities. Within the clusters, all the households with children ≤ 14 years of age were selected. In a household where only one child within that age was present, the child was selected automatically. In a case where more than one child was present in a household, only one was randomly selected. A probability proportionate to size sampling method was used to select 400 random malaria negative samples from all the negative samples in the study population for the sub-microscopic studies.

### Collection of data

Sociodemographic data which included information on gender, age, literacy level of parents/caregivers and malaria preventive methods, as well as fever history of participants were collected using a structured questionnaire. Axillary temperature was measured using an electronic thermometer with a febrile condition considered as temperature ≥ 37.5 °C [26].

### Laboratory methods

Three to four (3–4) millilitres (mL) of venous blood sample was collected from each child using sterile disposable syringes. Part of the blood sample was used to prepare thick and thin films on the same slide for the determination of the presence of malaria parasite by Giemsa-stain microscopy using standard methods [27]. Parasite densities were expressed as asexual parasites per µL with reference to the participant’s white blood cell count (WBC) and categorized as low, moderate, high and hyper parasitaemia [28].

Also, 50 μL of the EDTA blood sample was aliquoted onto a Whatman 3 mm filter paper and dried overnight at room temperature. The dried blood spots (DBS) were used to determine sub-microscopic *Plasmodium* infection. Genomic deoxyribonucleic acid (DNA) was isolated from the DBS using chelex [29]. Primary and nested polymerase chain reaction (PCR) assays were carried out for all genes. The primary PCR was carried out with a pair of *Plasmodium* genus-specific primers (rPLU5-5ʹCCTGTTGTTGCCTTAAACTTC3ʹ and rPLUS6-5ʹTTAAAATTGTTGCAGTTAAAACG3ʹ) which amplified a 1100-base pair (bp) PCR product from the rRNA small subunit gene (18S rRNA) while the nested primers specific for *P. falciparum* (rFAL1-5ʹTTAAACTGGTTTGGGAAAACCAAATATATT3ʹ) and rFal2-5ʹACACAATGAACTCAATCATGACTACCCGTC3ʹ) were used, which amplified a 205-bp indicating a *P. falciparum* infection [30].

Furthermore, an auto-haematology analyser (Urit-3300® analyser, Guangxi, China) was used to assess haematological parameters following the manufacturer’s instructions and the condition of anaemia was defined as haemoglobin level (Hb) < 11 g/dL of whole blood [27].

### Definitions and end points

- Sub-microscopic infection was defined as low-density blood-stage malaria parasite infection that was not detected by conventional microscopy but positive using PCR.
- Asymptomatic malaria parasitaemia was defined as the presence of *Plasmodium* by microscopy and with an axillary temperature of < 37.5 °C and no record of fever within the past 2 weeks.
- Parasitaemia was categorized as low (< 1,000 parasites/μL blood), moderate (1,000‒4,999 parasites/ μL blood), high (5000‒99,999 parasites/ μL blood), and hyperparasitaemia (≥ 100,000 μL) [28].
- Anaemia was defined as Hb < 11.0 g/dL and further categorized as severe (Hb < 7.0 g/dL), moderate (Hb between 7.0 and 10.0 g/dL), and mild (Hb between 10.1 and < 11 g/dL) [26].

### Statistical analysis

Continuous variables were summarized into means and standard deviations (SD) and categorical variables reported as frequencies and percentages, were used to evaluate the descriptive statistics. The differences in proportions were evaluated using Pearson’s Chi-Square (χ^2^). Group means were compared using Kruskal Wallis and Mann–Whitney U Test. Parasite densities were log transformed before analysis. Associations between predictor variables and primary outcomes were assessed using both bivariate and multivariate logistic regression analysis. Odd ratios (ORs) and 95% confidence intervals (CIs) were computed. Any covariate with a P value < 0.2 in the bivariate analysis was subsequently included in the final multivariable logistic model. Significant levels were measured at 95%CI with the level of significance set at P < 0.05. Post entry and clean-up of data in Microsoft Excel 2016, analysis was performed using the IBM-Statistical Package for Social Sciences (IBM-SPSS) version 20 and Epi-info version 7.

## Results

### Socio–demographic and clinical characteristics of the study population

A total of 1319 children with a mean (SD) age of 6.0 (3.5) years, residing at lowland (30.7%, 405), middle belt (37.2%, 491) and highland (32.1%, 423) in the Mount Cameroon area were evaluated. As shown in Table 1, most of the parents/caregiver of the children had a primary (47.9%) and secondary (31.6%) level of education. The proportion of febrile children in the study population was 8.5% (112), with no significant differences in age and sex. The prevalence of malaria parasite, asymptomatic malaria parasitaemia and sub-microscopic infection in the study population was 36.4%, 34.0% and 43.8%, respectively. Children between 5 and 9 years had the highest occurrence of microscopic (39.4%) and asymptomatic parasitaemia (36.9%) at P = 0.021 and P = 0.047 respectively, when compared with their contemporaries.

**Table 1:** Demographic, altitude and clinical characteristics of the participants by age and sex

Parameter	Age groups in years			Gender		Total
	< 5	5–9	10–14	Male	Female	
% (N)	38.1 (503)	42.2 (557)	19.6 (259)	49.4 (652)	50.6 (667)	100 (1319)
Mean age (SD) in years	2.5 (1.2)	6.7 (1.4)	11.5 (1.2)	6.3 (3.5)	5.9 (3.5)	6.0 (3.5)
Mean haemoglobin (SD) level in g/dL	9.9 (2.0)	10.6 (1.7)	11.2 (1.7)	10.4 (1.8)	10.5 (1.9)	10.5 (1.8)
Educational level of parent/caregiver						
No formal (n)	8.9 (42)	11.9 (57)	7.2 (16)	10.5 (61)	9.2 (54)	9.8 (115)
Primary (n)	42.9 (202)	53.2 (255)	47.1 (104)	45.4 (264)	50.4 (297)	47.9 (561)
Secondary (n)	37.4 (176)	23.8 (114)	36.2 (80)	31.6 (184)	31.6 (186)	31.6 (370)
Tertiary (n)	10.8 (51)	11.1 (53)	9.5 (21)	11.5 (73)	8.8 (52)	10.7 (125)
Altitude of residence						
Highland (n)	27.2 (137)	32.8 (178)	41.7 (108)	53.7 (227)	46.3 196)	32.1 (423)
Middle belt (n)	42.5 (214)	38.7 (215)	23.9 (62)	44.4 (218)	55.6 (273)	37.2 (491)
Lowland (n)	30.4 (153)	29.3 (163)	34.4 (89)	51.1 (207)	48.9 (198)	30.7 (405)
Clinical						
Fever prevalence (n)	8.3 (42)	9.5 (53)	6.6 (17)	8.1 (53)	8.8 (59)	8.5 (112)
Microscopic malaria parasite prevalence (n)	36.7 (185)^α^	39.4 (219)^α^	29.3 (76)^α^	36.0 (235)	36.7 (245)	36.4 (480)
Asymptomatic malaria parasite prevalence (n)	34.0 (164)^β^	36.9 (197)^β^	28.0 (71)^β^	33.6 (211)	34.4 (221)	34.0 (432)
Sub microscopic infection prevalence (n)	28.7 (47)^γ^	51.3 (79)^γ^	60.5 (49)^γ^	46.9 (91)	40.8 (84)	43.8 (175)

^α^ Significant difference with age (χ^2^ = 7.651 P = 0.021)

^β^ Significant difference with age (χ^2^ = 6.130 P = 0.047)

^γ^ Significant difference with age (χ^2^ = 27.358 P < 0.001)

^ε^ Significant difference with age (χ^2^ = 34.428 P < 0.001)

Fever = axillary temperature ≥ 37.5ºC

The prevalence of sub microscopic malaria was significantly highest (P < 0.001) among the 10–14 years age group (60.5%) when compared with the other age groups (Table 1).

### Asymptomatic malaria prevalence by altitude

**Fig. 2:**
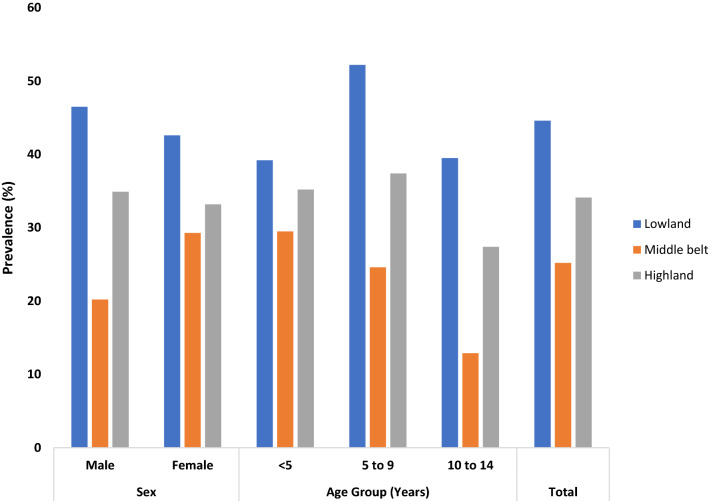
Effect of age and sex on asymptomatic malaria prevalence stratified by altitude in the study population

The overall microscopic prevalence of malaria parasite was 36.4% (CI: 33.8–39.0). The prevalence of asymptomatic falciparum malaria among the 1271 children without fever varied with altitude. The overall prevalence in the low, middle belt and high lands was 44.6% (CI: 39.8–49.6), 25.2% (CI: 39.8–49) and 34.1% (CI: 29.6–38.8), respectively, and the difference was statistically significant (χ^2^ = 35.980, P < 0.001) (Fig. 2). In the lowland, asymptomatic malaria was significantly highest (χ^2^ = 6.651, P = 0.036) in children aged 5‒9 years (52.2%, CI: 44.5–59.9) old while, in the middle belt it was significantly highest (χ^2^ = 7.007, P = 0.03) in children < 5 years (29.5%, CI: 23.7–36.0) when compared with their respective counterparts. No significant difference with age was observed in highlands even though the prevalence was highest in children 5–9 years old (37.4%, CI: 30.5–44.9). On the other hand, while children < 5 years in the low, middle belt and high lands had similar prevalence of asymptomatic malaria parasitaemia, those 5–9 years (52.2%, CI: 44.5–59.9) and 10–14 years (39.5%, CI: 29.9–50.1) resident in the lowlands, had the highest prevalence and the difference was significant (χ^2^ = 28.830, P < 0.001 and χ^2^ = 12.720, P = 0.002, respectively). As shown in Fig. 2, asymptomatic malaria prevalence among the sexes was comparable within the low and high lands but statistically different in the middle belt (χ^2^ = 5.157, P = 0.023) where, females had higher prevalence (29.3%, CI: 24.1–35.0) than males (20.2%, CI: 15.4–26.1). Conversely, significantly higher (χ^2^ = 32.251, P < 0.001 and χ^2^ = 8.896, P = 0.012) prevalence was observed in males (46.5%, CI: 39.7–53.4) and females (42.6%, CI: 35.8–49.7) in the lowland when compared with those in the middle belt and highland, respectively.

### Malaria parasite density and category

The geometric mean parasite density (GMPD) was significantly higher (P < 0.001) in children residing in the lowland (449 parasites/ µL of blood) those < 5 years old in the lowland (538 parasites/ µL of blood, P = 0.024) and middle belt (224 parasites/ µL of blood, P = 0.003) and males (218 parasites/ µL of blood, P = 0.025) in the middle belt (see Additional file 1).

**Fig.3:**
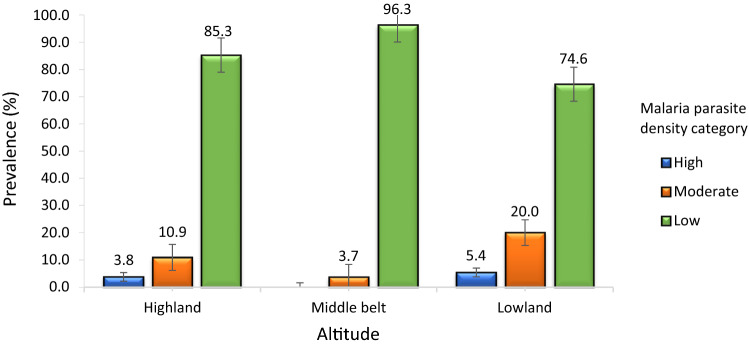
Prevalence of malaria parasite density category by altitudinal site

The prevalence of low, moderate and high parasitaemia in the study population were 84.2% (401/476), 12.4% (59/476) and 3.4% (16/476). As shown in Fig. 3, the prevalence of low, moderate and high malaria parasitaemia was significantly different (P < 0.001) in children from the different altitudes, with the low parasite density category being the most prevalent in all the three settings.

### Sub-microscopic infection prevalence and altitude

Overall, the prevalence of sub-microscopic malaria parasitaemia with respect to altitude was highest in children in the highland (66.7%) and lowest in the lowland (29.2%). With respect to age related differences and altitude, among children < 5 years, those resident in middle belt had the highest sub-microscopic malaria parasite prevalence (34.1%). On the other hand, the 5–9 and 10–14 years old, resident in highland had significantly higher (P < 0.001) prevalence (87.2% and 81.8%, respectively) than those in the other altitudinal sites. Moreover, males and female’s resident in the highlands had the highest prevalence of sub-microscopic infection (75.4% and 58.7%), compared to the other altitudinal sites at P < 0.001 and P = 0.002, respectively, as shown in Table 2.

**Table 2:** Sub-microscopic malaria parasite prevalence in relation to age and sex stratified by altitude

Parameter		Altitude						χ^2^; P
		Highland % (n)		Middle belt % (n)		Lowland % (n)		
Age (years)	< 5	40	30.0 (12)	82	34.1 (28)	42	16.7 (7)	4.197; 0.123
	5–9	47	87.2 (41)	59	40.7 (24)	50	30.0 (15)	36.036; < 0.001***
	10–14	33	81.8 (27)	19	42.1 (8)	28	46.4 (13)	11.229; 0.004**
χ^2^ P			36.556 < 0.001***	0.820 0.664		7.232 0.027*		–
Gender	Male	57	75.4 (43)	78	44.9 (35)	59	22.0 (13)	33.418, 0.001***
	Female	63	58.7 (37)	82	30.5 (25)	61	36.1 (22)	12.564; 0.002**
Total		120	66.7 (80)	160	37.5 (60)	120	29.2 (35)	38.519, 0.001***
χ^2^ P		3.759 0.053		3.529 0.060		2.858 0.91		–

* Statistically significant at P < 0.05** statistically significant at P < 0.01*** statistically significant at P < 0.001

### Anaemia prevalence and its severity

The overall prevalence of anaemia was 62.3%. No significant differences were observed with sex and febrile status while, the prevalence of anaemia decreased significantly (P < 0.001) with an increase in age with youngest age group having a prevalence of 73.8%. The occurrence of anaemia was significantly higher in children from the middle belt (72.7%), those with asymptomatic microscopic (68.1%) and sub-microscopic (78.3%) *Plasmodium* infection than their respective equal (Table 3). Relating to the severity of anaemia, children aged < 5 years had the highest prevalence of severe (7.8%) and moderate (58.8%) anaemia while mild anaemia was most common in those 10‒14 years old (53.4%) and the difference was statistically significant at P < 0.001. Significantly (P < 0.001 and P = 0.006), moderate anaemia was the most occurring form of anaemia in children negative for asymptomatic malaria parasitaemia (56.7%) and those positive for sub-microscopic infection (64.2%), respectively, as shown in Table 3.

**Table 3:** Prevalence and severity of anaemia as affected by altitude, age, sex, asymptomatic malaria, sub microscopic infection and febrile status

Variable	Category	No. examined	Anaemia prevalence % (n)	Anaemia severity prevalence			
				No. examined	Severe % (n)	Moderate % (n)	Mild % (n)
Altitude	Lowland	423	54.6 (221)	221	3.2 (7)	54.3 (120)	42.5 (94)
	Middle belt	405	72.7 (357)	357	3.9 (14)	54.1 (193)	42.0 (150)
	Highland	491	57.7 (244)	244	7.8 (19)	53.7 (131)	38.5 (94)
χ^2^ P		36.804, < 0.001***		6.828, 0.145			
Sex	Male	652	62.4 (407)	407	54.4 (22)	54.3 (221)	40.3 (164)
	Female	667	62.2 (415)	415	4.3 (18)	53.7 (223)	41.9 (174)
χ^2^ P		0.006 0.939		0.627 0.731			
Age group (Years)	< 5	503	73.8 (371)	371	7.8 (29)	58.8 (218)	33.4 (124)
	5–9	557	62.5 (348)	348	2.3 (8)	52.0 (181)	45.7 (159)
	10–14	259	39.8 (103)	103	2.9 (3)	43.7 (45)	53.4 (55)
χ^2^ P		84.121 < 0.001***		26.885 < 0.001***			
Asymptomatic malaria status	Positive	432	68.1 (294)	294	4.1 (12)	50.0 (147)	45.9 (135)
	Negative	839	58.0 (487)	487	5.3 (26)	56.7 (276)	38.0 (185)
χ^2^ P		12.062 < 0.001***		84.121 < 0.001***			
Sub-microscopic status	Positive	175	78.3 (137)	137	4.4 (6)	64.2 (88)	31.4 (43)
	Negative	225	43.1 (97)	97	6.2 (6)	43.3 (42)	50.5 (49)
χ^2^ P		50.167 < 0.001***		10.127 < 0.006**			
Febrile status	Febrile	112	67.0 (75)	75	2.7 (2)	62.7 (47)	34.7 (26)
	Afebrile	1207	61.9 (747)	747	5.1 (38)	53.1 (397)	41.8 (312)
χ^2^ P		1.124 < 0.289		2.800 0.247			

** Statistically significant at P < 0.01 *** statistically significant at P < 0.001

### Sub-microscopic infection and haematological indices

The mean haematological parameters were comparable between children with and without sub microscopic infection except for the mean Hb levels, haematocrit (Hct), RBC (red blood cell) and platelet (Plt) counts, mean corpuscular haemoglobin concentration (MCHC) and red cell distribution–coefficient of variation (RDW-CV). Children with sub microscopic infection had a significantly lower mean Hb concentration (9.86 ± 1.7 g/dL), RBC (4.48 ± 1.1 × 10^12^/L) and Plt (280.83 ± 112.62) counts, Hct (31.92%) and MCHC (31.33 ± 4.74 g/L) than their negative counterparts as shown in Table 4. On the other hand, the mean RDW-CV% (15.19 ± 3.3) was significantly higher (P < 0.001) in children with sub-microscopic infection than those negative.

**Table 4:** A comparison of mean haematological values in children positive for sub-microscopic infection and those negative

Variable	Sub microscopic status	Mean (SD)	t-test	95% CI of difference
			P value	
WBC × 10^9^/L	Pos	7.55 (2.87)	0.22	− 0.46 to − 0.58
	Neg	7.48 (2.40)	0.824	
Hb (g/dL)	Pos	9.86 (1.65)	− 6.87	− 1.69 to − 0.94
	Neg	11.18 (2.07)	< 0.001***	
RBC × 10^12^/L	Pos	4.48 (1.05)	− 4.61	− 0.76 to − 0.31
	Neg	5.01 (1.22)	< 0.001***	
Hct (%)	Pos	31.92 (7.16)	3.33	− 4.31 to − 1.11
	Neg	33.63 (8.74)	< 0.001***	
MCV (fl)	Pos	72.58 (8.84)	1.40	− 0.45 to − 2.68
	Neg	71.46 (7.10)	0.163	
MCH (pg)	Pos	34.16 (3.36)	2.15	− 1.14 to − 0.3
	Neg	36.68 (3.83)	0.253	
MCHC (g/L)	Pos	31.33 (4.74)	− 2.11	− 21.64 to − 21.63
	Neg	32.45 (5.64)	0.035	
RDW-CV%	Pos	15.19 (3.29)	3.24	0.38 to − 1.56
	Neg	14.21 (2.71)	< 0.001***	
Plt × 10^9^/L	Pos	280.63 (112.62)	− 2.66	− 48.61 to − 0.84
	Neg	305.36 (126.32)	0.049*	

POS: positive, Neg: negative, * Statistically significant at P < 0.05*** statistically significant at P < 0.001, Pos = 175, Neg = 225

### Risks factors of sub-microscopic *Plasmodium* infection

The logistic regression model with sub-microscopic infection status as dependent variable and altitude, age, gender, marital status, ITN usage, fever, fever within a month, anaemia and water source as independent variable, demonstrated that children from highlands (P =  < 0.001), those between 5 and 9 years (P =  < 0.001) and 10‒14 years (P =  < 0.001), those who did not use ITN (P = 0.04) and anaemic children (P =  < 0.001) were more likely to have sub-microscopic malaria parasite infection. The odds of carrying sub-microscopic infection is presented in Table 5. Children from highlands, those 5‒9 years and between 10 and 14 years of age, who did not use ITN and anaemic as well were 1.8, 3, 8, 1.69 and 9 times greater the odds to carry sub-microscopic *Plasmodium* infection than their counterparts.

**Table 5:** Logistic regression model examining factors associated with sub-microscopic *Plasmodium falciparum* infection in the study population

Variables	N	Sub-microscopic infection prevalence (n)	Bivariate logistic regression		Multivariate logistic regression	
			COR (95% CI)	P value	AOR	P value
Altitude						
Lowland	120	29.2 (35)	Reference		Reference	
Middle belt	160	37.5 (60)	1.46 (0.88–2.42)	0.15	0.52 (0.25–1.08)	0.08
Highland	120	66.7 (80)	4.86 (2.81–8.39)	< 0.001***	1.76 (0.86–3.60)	0.13
Age group (Years)						
< 5	165	28.5 (47)	Reference		Reference	
5–9	154	51.3 (79)	2.64 (1.66–4.20)	< 0.001***	3.13 (1.77–5.56)	< 0.001***
10–14	81	60.5 (49)	3.84 (2.20–6.72)	< 0.001***	8.18 (3.91–17.20)	< 0.001***
Gender						
Male	195	47.2 (92)	Reference	–		
Female	205	40.5 (83)	0.76 (0.51–1.13)	0.18	0.74 (0.45–1.20)	0.22
Marital status						
Married	293	40.6 (119)	Reference	–		
Single	97	51.5 (50)	1.56 (0.98–2.47)	0.06	1.67 (0.94–2.96)	0.08
Use of ITN						
Yes	221	35.7 (79)	Reference	–		
No	179	53.6 (96)	2.08 (1.39–3.11)	< 0.001***	1.69 (1.01–2.81)	0.04*
Fever						
Yes	28	32.1 (9)	Reference			
No	372	44.6 (166)	1.7 (0.75–3.86)	0.20	2.21 (0.77–6.40)	0.14
Fever within a month						
No fever	272	42.6 (116)	Reference		–	–
Fever	128	46.1 (59)	1.15 (0.75–1.76)	0.52	–	–
Anaemia						
No	166	22.9 (38)	Reference			
Yes	234	58.5 (137)	4.76 (3.05–7.43)	< 0.001***	9.01 (4.51–17.99)	< 0.001***
Malnourished						
No	271	42.8 (116)	Reference	–	–	–
Yes	129	45.7 (59)	1.13 (0.74–1.72)	0.58	–	–
Water source						
Close	357	42.6 (152)	Reference	–	Reference	–
Open	43	53.5 (23)	1.55 (0.82–2.93)	0.18	1.28 (0.59–2.75)	0.53
Stunting						
No	309	41.7 (129)	Reference			
Yes	91	50.5 (46)	1.43 (0.89–2.28)	0.14	1.54 (0.85–2.80)	0.15

AOR: adjusted odd ratio, COR: crude odd ratio, *Statistically significant at P < 0.05, *** statistically significant at P < 0.001

## Discussion

Considerable progress has been made in the past years in reducing malaria morbidity and mortality in Africa, with Cameroon inclusive, largely due to interventions such as LLIN and use of artemisinin-based combination therapy [21, 31]. Detailed assessments of parasite carriage by conventional diagnostics alongside molecular investigation have uncovered that a considerable proportion of malaria infections is undetected by routine microscopy [32]. In settings where recent malaria control efforts have been successful, and across various endemicities, sub-microscopic infections frequently outnumber microscopically detectable infections [5, 16, 33]. Although high levels of sub-microscopic infection occur in many different settings [19, 33–35], studies on their clinical significance are limited and further insight is necessary for proper morbidity management in malaria endemic areas. This cross-sectional study examines the influence of asymptomatic and sub-microscopic *P. falciparum* infection on anaemia and haematological indices as public health problems in children ≤ 14 years across low, middle belt and highland altitudes in the Mount Cameroon area.

Findings from the study suggests that children < 5 years and 5–9 years in the middle belt and lowland respectively, are the most affected by the malaria parasite and, therefore, constitute sensitive groups for monitoring changes in malaria burden using microscopy in the Mount Cameroon area. Case management which is one of the current surveillance methods in the country focus more on the < 5 years age group, although findings from this study indicated that 5–9 years old had more detectable infections. Consequently, health education and treatment should not only target vulnerable groups (< 5 years and pregnant women), but all the age groups.

The prevalence and density of asymptomatic malaria parasitaemia with respect to age and gender were significantly different across the different altitudinal sites. This result is not surprising because several studies have reported that in Cameroon, malaria burden and transmission intensity are heterogeneous with spatial and temporal variations between altitudes and geographical areas, with prevalence rates varying from one area to another [4]. Although the prevalence of asymptomatic malaria parasite was comparable between males and females, the density was however higher in males than in females among middle belt dwellers. This is in line with an earlier study by Kimbi et al. [36] and Sumbele et al. [21]. In addition, the effect of gender on the outcome of *P. falciparum* infection has previously been reported in other parts of Africa [37, 38]. Hormonal differences between the sexes may also be a contributing factor to the difference in malaria parasite prevalence. Cernetich et al. [39] showed that synthesis of testosterone by males suppresses antiplasmodial immune response, whereas production of oestrogen augments antiplasmodial immune response.

The present study is the first large-scale description of sub-microscopic malaria parasite prevalence among children in three communities in the Mount Cameroon area using the nested PCR method. The overall sub-microscopic malaria parasitaemia of 43.8% was observed in microscopic negative slides by PCR in the study population. In line with other studies, Okell et al. [15] reported that the proportion of sub-microscopic infections is inversely correlated with slide prevalence and parasite density on the global level. Bousema et al. [5] reported that individuals with sub-microscopic malaria parasite are accountable for maintaining *Plasmodium* species between transmission season, since they are a major reservoir.

Findings from the study indicated that the proportion of sub-microscopic infection in the communities were significantly associated with age, as older children had an increased chance of being carriers of sub-microscopic infection compared with those younger. This is consistent with reports from other studies from Uganda [40], Kenya [41], India [42] and Ethiopia [43] who reported that older children do not easily develop symptomatic malaria upon infection both because they tolerate parasite densities better without developing fever and because they are at lower risk to develop high parasite densities. Age is a key factor that correlates positively with protective immunity in malaria-endemic areas. It has been reported that parasitaemia in older age groups is lower than the detection limits of conventional malaria diagnostic tools, which therefore fail to detect parasitaemia [44].

The importance of the sub-microscopic parasite pool rests on the understanding that sub-microscopic infections can transmit malaria [45], although the minimum parasite density necessary for transmission is unknown. Worthy of note in the Mount Cameroon area, sub-microscopic *Plasmodium* infections in older children may be an important source of the local transmission of the parasite. In addition, the unusual significantly higher GMPD observed in children < 10 years living in the highlands than those in the middle belt is also of concern especially as the climatic conditions in the highlands are considered unfavourable for the development of the vector and transmission of the parasite.

The higher prevalence of sub-microscopic infection in highland dwellers than lowland support findings of other studies [14, 46], which suggests that the burden of sub-microscopic infections is highly heterogeneous across different locations. Although several hypotheses could account for this, one possible explanation might be differences in the extent of parasite genetic diversity between settings [47–49]. In low transmission settings, repeated exposure to a limited number of strains might lead to rapid development of protective immunity against those strains. Individuals in these settings would then be expected to have, on average, a higher proportion of infected sub-microscopic population. By contrast, in high transmission settings, higher circulating parasite genetic diversity would mean that individuals are more frequently infected with strains they have not previously encountered. However, in contrast, a recent characterisation of sub-microscopic malaria carriage at three Ugandan sites with varied transmission intensity revealed little change in the extent and size of the sub-microscopic reservoir across the transmission gradient at the sub-national level [13].

In addition to altitudinal effect that may affect transmission dynamics, findings from the study revealed non-users of ITN were 2-folds more likely to carry sub-microscopic infection. This observation supports the findings of other studies that proper use of ITN significantly reduces malaria morbidity and mortality [2, 50].

It has been reported that sub-microscopic infections go undetected and untreated with little or no clinical manifestation in many malaria endemic communities [51]. However, findings from this study demonstrated that these infections are associated with anaemia as well, a decrease in some red cell indices and platelet counts. Anaemic children were 9 times more likely to carry sub-microscopic infection when compared to non-anaemic children, demonstrating the clinical relevance of sub-microscopic infection. The result agrees with studies by Rek et al. [13] and De Mast et al. [35] who also suggested an association between sub-microscopic malaria infection and anaemia. Anaemia is multifactorial in aetiology, however observations from this study enriches the body of evidence suggesting the detrimental clinical consequences of parasitaemia of any density [17, 33].

The high prevalence of anaemia (62.3%) in children less than or equal to 14 years among the population in this area highlights anaemia as a severe public health problem in malaria endemic communities. The association between malaria parasitaemia and anaemia is well established in previous studies [26, 36, 52–54]. Malaria parasitaemia causes devastation of parasitized and non-parasitized red blood cells hence reducing haemoglobin levels leading to anaemia. The higher proportion of anaemia in the younger age group is in line with previous studies that anaemia due to malaria is more severe in younger children in areas of intense transmission [55, 56]. Children in this age group are more vulnerable to infection with malaria than others with severe and potentially fatal complications.

Sub-microscopic *Plasmodium* infection in the study was associated with lower Hb, Hct, RBC count as well as MCHC as confirmed by the decrease in their mean values in those positive. It is most likely that sub-microscopic *Plasmodium* infection would have exacerbated the reduction in the red cell indices as asymptomatic parasitaemia and protracted malaria infections have been associated with a marked reduction in Hb concentration and with a clinically significant RBC destruction [57] indicating that parasitological cure is necessary for haematological recovery [58].

Findings revealed reduction in platelet count in children with sub-microscopic *Plasmodium* infection. The association of platelet count and malaria has previously been described [59]. However, the reduction did not culminate in thrombocytopenia, which is the reduction in platelet count below the normal range (150,000—450,000 µL of blood) that has been postulated as a marker of *Plasmodium* infection. Thrombocytopenia seems to occur through peripheral destruction [60], excessive removal of platelet by spleen pooling [61] as well as platelet consumption by the process of disseminated intravascular coagulopathy. Also, immune-mediated destruction of circulating platelets has been postulated as a cause of thrombocytopenia [62].

While the findings reported have implications for the control and better management of malaria related morbidities in the Mount Cameroon area it could have a wider applicability in other regions with similar altitudinal ranges and environmental conditions. The study is however not without limitation, the study design does not allow the assessment of causality between asymptomatic and sub microscopic parasitaemia and other aetiologies associated with anaemia. However, the findings add invaluable evidence to the clinical relevance of sub-microscopic infection and contributions of asymptomatic malaria to the burden of anaemia, which is often overlooked.

## Conclusions

The significant heterogeneity in the burden of asymptomatic and sub-microscopic *Plasmodium* infection in addition to its corollary on haematological variables among children in the different attitudinal sites of the Mount Cameroon Region accentuate the need for strategic context specific planning of malaria control and preventative measures. While proper case management continues to be a focus of control efforts, novel strategies are also needed to target the asymptomatic and sub-microscopic parasite reservoirs alongside the consequences on anaemia and haematological indices among children in endemic regions. This information is priceless to use the limited resources in a cost-effective way to appropriately implement management.

## Supplementary Information

**Additional file 1.** Although not significant GMPD decreased with an increase in age in the high lands as well as the higher values observed in males than females in the low (465) and high lands (385). However, the GMPD/ µL of blood in males (465) and females (434) was significantly higher (P < 0.001) in the lowland when compared with the other altitudinal sites.

## Data Availability

All datasets on which the conclusions of the research rely are presented in this paper. However, data is available from the corresponding author on reasonable request.

## Abbreviations

AOR: Adjusted odd ratio
asl: Above sea level
ACT: Artemisinin-based combination therapy
CI: Confidence interval
COR: Crude odd ratio
DBS: Dried blood spot
DNA: Deoxyribonucleic acid
EDTA: Ethylenediaminetetraacetate
GMPD: Geometric mean parasite density
Hb: Haemoglobin
Hct: Haematocrit
ITNs: Insecticide-treated bed nets
LLINs: Long-lasting insecticidal nets
MA: MAlaria anaemia
MCH: Mean corpuscular haemoglobin
MCHC: Mean corpuscular haemoglobin concentration
MCV: Mean corpuscular volume
OR: Odd ratio
PCR: Polymerase chain reaction
Plt: Platelet
RBC: Red blood cell
SD: Standard deviations
RDW-CV: Red cell distribution-coefficient of variation
WHO: World Health Organization
