# Supplementary material for: Asymptomatic and sub-microscopic Plasmodium falciparum infection in children in the Mount Cameroon area: a cross-sectional study on altitudinal influence, haematological parameters and risk factors
Source: Malar J. 2021 Sep 26;20:382. doi: 10.1186/s12936-021-03916-7 (PMC8474836; doi:10.1186/s12936-021-03916-7)
Supplement: Supplementary file 1 — Additional file 1. Although not significant GMPD decreased with an increase in age in the high lands as well as the higher values observed in males than females in the low (465) and high lands (385). However, the GMPD/ µL of blood in males (465) and females (434) was significantly higher (P < 0.001) in the lowland when compared with the other altitudinal sites. [file 12936_2021_3916_MOESM1_ESM.docx]

**Table 2:** Malaria parasite density in the different age groups and sex at different altitudes

| Parameter | | Altitude | | | P value |
| --- | --- | --- | --- | --- | --- |
|  |  | Lowland GMPD/ µL of blood (CI) | Middlebelt GMPD/ µL of blood (CI) | Highland GMPD/ µL of blood (CI) |  |
| Age | < 5 | 538 (207.2 – 868.8) | 224 (188.0 – 260.0) | 399 (248.1 – 1046.1) | < 0.001^a^ |
|  | 5–9 | 456 (158.9 – 753.1) | 158 (129.8 – 186.2) | 379 (132.3 – 890.3) | < 0.001^a^ |
|  | 10–14 | 320 (3.4 – 636.6) | 142 (119.9 – 164.1) | 321 (182.7 – 459.3) | 0.078 |
| P value | | 0.024*^a^ | 0.003**^a^ | 0.736 | - |
| Sex | Male | 465 (203.3 – 726.7) | 218 (181.9 – 254.0) | 385 (164.1 – 932.1) | 0.001^a^ |
|  | Female | 434 (169.7 – 698.3) | 145.9 (80 – 198.1) | 174.3 (174.3 – 545.7) | < 0.001^a^ |
| Total | | 449 (100 – 11520) | 449 (263.9 – 634.0) | 187 (165.7 – 208.3) | 374 (63.1 – 684.9) |
| P- value | | 0.563 | 0.025*^b^ | 0.728 | - |

*Statistically significant at P < 0.05 ** statistically significant at P < 0.01 *** statistically significant at P < 0.001.

^a^Difference in GMPD in the different altitude and age groups determined by Kruskal–Wallis test

^b^Difference in GMPD in the different sex determined by Mann–Whitney U test

CI = 95% confidence interval of the geometric mean
